# De Novo Mutation Rate Estimation in Wolves of Known Pedigree

**DOI:** 10.1093/molbev/msz159

**Published:** 2019-07-12

**Authors:** Evan M Koch, Rena M Schweizer, Teia M Schweizer, Daniel R Stahler, Douglas W Smith, Robert K Wayne, John Novembre

**Affiliations:** 1 Department of Ecology and Evolution, University of Chicago, Chicago, IL; 2 Division of Biological Sciences, University of Montana, Missoula, MT; 3 Department of Ecology and Evolutionary Biology, University of California, Los Angeles, Los Angeles, CA; 4 Department of Biology, Colorado State University, Fort Collins, CO; 5 Yellowstone Center for Resources, National Park Service, Yellowstone National Park, WY; 6 Department of Human Genetics, University of Chicago, Chicago, IL

**Keywords:** demographic history, mutation rate, dog domestication

## Abstract

Knowledge of mutation rates is crucial for calibrating population genetics models of demographic history in units of years. However, mutation rates remain challenging to estimate because of the need to identify extremely rare events. We estimated the nuclear mutation rate in wolves by identifying de novo mutations in a pedigree of seven wolves. Putative de novo mutations were discovered by whole-genome sequencing and were verified by Sanger sequencing of parents and offspring. Using stringent filters and an estimate of the false negative rate in the remaining observable genome, we obtain an estimate of ∼4.5 × 10^−9^ per base pair per generation and provide conservative bounds between 2.6 × 10^−9^ and 7.1 × 10^−9^. Although our estimate is consistent with recent mutation rate estimates from ancient DNA (4.0 × 10^−9^ and 3.0–4.5 × 10^−9^), it suggests a wider possible range. We also examined the consequences of our rate and the accompanying interval for dating several critical events in canid demographic history. For example, applying our full range of rates to coalescent models of dog and wolf demographic history implies a wide set of possible divergence times between the ancestral populations of dogs and extant Eurasian wolves (16,000–64,000 years ago) although our point estimate indicates a date between 25,000 and 33,000 years ago. Aside from one study in mice, ours provides the only direct mammalian mutation rate outside of primates and is likely to be vital to future investigations of mutation rate evolution.

## Introduction

Understanding the rates and biochemical sources of new mutations is of inherent interest to evolutionary biologists. New germline mutations provide genetic variation from which many new adaptations are built, but they also may incur a fitness cost to organisms when they are deleterious (Agrawal and Whitlock 2012). The rate of mutation plays a crucial role in calibrating molecular clocks and allowing branch lengths of genealogies to be converted to units of time (Kimura 1968; Bromham and Penny 2003). In demographic inference, any uncertainty in the mutation rate is directly propagated to estimates of the divergence time, effective size, and migration between populations. In humans, for example, a recently revised reduction of the per-generation mutation rate by a factor of 2 had profound impact on the timing of all major evolutionary transitions, from our divergence with chimpanzees to the time the first modern humans left Africa (Scally and Durbin 2012; Ségurel et al. 2014).

One controversial issue concerning mutation rate is its impact on inferences of the timing and location of dog domestication. Genetic estimates of divergence time between dogs and the gray wolf (the wild ancestor of dogs), range from 14,000 to over 100,000 years before present (reviewed in Freedman, Lohmueller, et al. [2016] and Ostrander et al. [2017]). A seemingly straightforward approach to the study of dog domestication would be to fit a demographic model to extant dog and wolf populations (Freedman et al. 2014; Freedman, Schweizer, et al. 2016); the wolf population from which dogs split most recently would identify the geographic location of domestication, and the time of this split would give an accurate upper bound on the timing. Unfortunately, this conceptually simple approach is complex in practice. Inferring the geography of domestication requires that the modern descendants of the source wolf population are not too far from their ancestors’ location, and inferring the split time is strongly dependent on mutation rate.

A commonly used per base pair mutation rate for dogs and wolves has been 1 × 10^−8^ per generation (Lindblad-Toh et al. 2005; Freedman et al. 2014; Skoglund et al. 2015; for brevity here on we use “per base pair” in referring to the mutation rate). The value of 1 × 10^−8^ per generation is close to the value of 6.6 × 10^−9^ obtained if one multiples the average mammalian mutation rate of 2.2 × 10^−9^ per year measured by Kumar and Subramanian (2002) using substitution on a fossil-calibrated phylogeny by a generation time of 3 years (Lindblad-Toh et al. 2005; Skoglund et al. 2015). Given how little was known about the mutation rate in dogs, Freedman et al. (2014) considered the range of mutation rates from 6.6 × 10^−9^ to 1.8 × 10^−8^ per generation to provide a range for the split time between dogs and wolves to be between 11,000 and 34,000 years ago. Wang et al. (2013) also estimated the split time between dogs and wolves and used the mutation rate of 6.6 × 10^−9^ per generation. If they had used a rate of 1.8 × 10^−8^ instead their estimated split time would have been about 21,000 years ago. Much of the discordance in estimated split times was therefore due to different assumptions about the mutation rate.

Two recent studies have estimated the mutation rate specifically for dogs and wolves using ancient DNA. Skoglund et al. (2015) used an approach originally developed to estimate the divergence time between the ancestral populations of humans and Neanderthals (Green et al. 2010). The procedure uses the proportion of sites carrying the derived allele in the ancient individual, conditional on that site being heterozygous in the modern individual. If the demographic history of the population ancestral to the modern individual and the divergence times of the populations ancestral to the modern and ancient individual are known, then a mutation rate can be chosen to fit the observed data. Skoglund et al. (2015) applied this approach to ancient DNA from a 35,000-year-old wolf from the Taimyr Peninsula in Siberia. Frantz et al. (2016) applied a similar approach to a 4,800-year-old dog from the Newgrange site in Ireland but were able to fit a joint demographic history to both the ancient and modern samples because their ancient sample was of higher quality. These studies reported mutation rates of 4.0 × 10^−9^ and 3.0–4.5 × 10^−9^ per generation, respectively. Both rates are lower than those used previously and push the estimated divergence time between dogs and wolves further into the past. In particular, when calibrating their model using the mutation rate from Skoglund et al. (2015), Fan et al. (2016) estimate a divergence time around 29,000 years ago.

However, there are problems with all these approaches to mutation rate estimation. First, a demographic history is estimated using an approach such as the pairwise sequentially Markovian coalescent approach (Li and Durbin 2011), and it is not clear how deviations from the true population history and uncertainty in the distribution of coalescent times impact estimation (Beichman et al. 2018). Second, the age of the ancient specimen is used for the population split date, and this will give an overestimate because the ancient individual is likely not from the same population as the ancestors of the modern individual. For instance, the ancient Taimyr wolf population is unlikely to be directly ancestral to that of modern wolves. Additionally, the Green et al. (2010) approach assumes no postdivergence gene flow, a process known to occur in dogs and wolves since domestication (Freedman et al. 2014). The presence of gene flow would increase similarity between ancient and modern samples and lead to underestimation of the mutation rate.

To estimate mutation rate, we use a whole-genome sequencing approach of parents and offspring of wolves from Yellowstone National Park, USA. This approach is insensitive to the issues of fossil calibration and demographic assumptions surrounding previous calculations. Estimating the mutation rate by sequencing parents and offspring is conceptually straightforward and involves a count of the number of sites where both parents are homozygous for the same allele and the offspring is heterozygous divided by the number of observed sites in the genome. In practice, however, it can be difficult to distinguish true de novo mutations (DNMs) from sequencing errors, somatic mutations, missed heterozygous genotypes in parents, and alignment issues in repetitive regions of the genome. Nonetheless, pedigree-based estimation of mutation rates has been performed in a growing number of species. Estimates based on pedigree sequencing are available for *Homo sapiens* (Kong et al. 2012), *Pan troglodytes* (Venn et al. 2014; Besenbacher et al. 2019), *Pongo abelii* (Besenbacher et al. 2019), *Gorilla gorilla* (Besenbacher et al. 2019), *Drosophila melanogaster* (Keightley et al. 2014), *Heliconius melpomene* (Keightley et al. 2015), *Apis mellifera* (Yang et al. 2015), *Arabidopsis thaliana* (Yang et al. 2015), *Ficedula albicollis* (Smeds et al. 2016), *Chlorocebus pygerythrus* (Pfeifer 2017a), and *Aotus nancymaae* (Thomas et al. 2018). In humans, pedigree studies produce lower estimates of the mutation rate around 1.2 × 10^−8^ per generation (Ségurel et al. 2014) compared with 2.3 × 10^−8^ per generation, a value that had been calculated using a fossil-calibrated divergence time with chimpanzees. As mentioned above, this lower value suggests estimated times of demographic events would have to be increased by a factor of 2. However, an increase in the generation time could compensate for this decrease by reducing the average number of mutations occurring per year (Scally and Durbin 2012; Ségurel et al. 2014; Amster and Sella 2016).

**Fig. 1.**
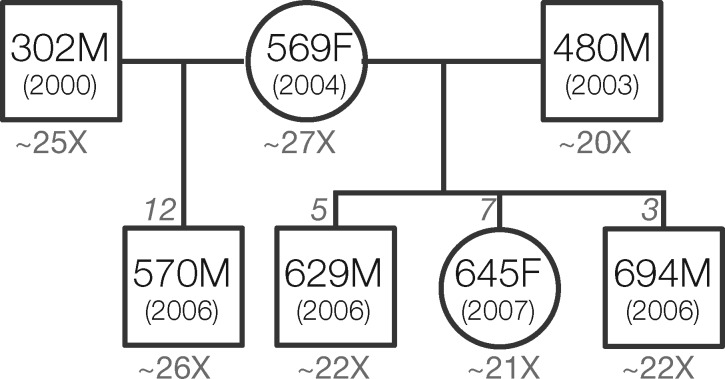
Whole-genome sequences from seven Yellowstone wolves of known pedigree were analyzed to detect DNMs. For each male (square) or female (circle) wolf, the top number indicates the Yellowstone National Park wolf ID and the bottom number provides the birth year. Below each individual is the average sequencing depth of coverage (see supplementary fig. S1, Supplementary Material online, for more detail on coverage per individual). The number of verified DNMs in each of the four offspring is provided at the top of the box or square.

In this study, we estimate the mutation rate in wolves by sequencing a pedigree that consists of four offspring, one mother, and two fathers from Yellowstone National Park (fig. 1). We identified DNMs by applying strict filters based on genomic context and independently verifying a large number of candidate sites using Sanger sequencing (following the general pipeline developed by Keightley et al. [2014]). Then, we calculated the posterior probability of different mutation rates based on the number of sites in the genome passing all filters and estimated false negative rates (FNRs). We bound the mutation rate between 2.6 × 10^−9^ and 7.1 × 10^−9^ per base pair per generation and give a point estimate of 4.5 × 10^−9^, which is consistent with previous estimates based on ancient DNA.

## Results

### Sequencing Filtering and Identification of DNMs

We identified DNMs using whole-genome sequencing data from a known pedigree of seven wolves (Schweizer et al. 2018; vonHoldt et al. 2008) (fig. 1). Interestingly, this pedigree family structure reveals the first confirmed case of multiple paternity of a single litter in gray wolves in the wild. In 2006, Wolf 569F was a subordinate breeding female of the Druid Peak Pack and produced a litter containing genotyped offspring of both subordinate breeding male 302M (offspring 570M) and dominant breeding male 480M (offspring 629M and 694F).

**Fig. 2.**
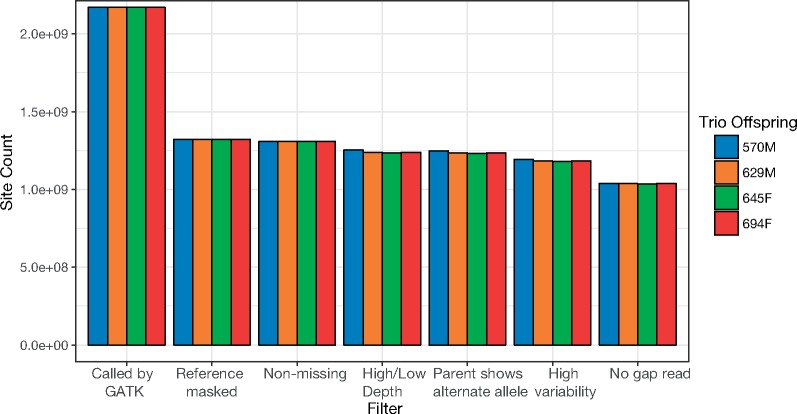
Number of sites remaining after the sequential application of filters. Filters were applied independently in each trio to remove regions of the genome producing false positives. The final bars represent the number of sites ultimately examined for candidate DNMs. Filters were applied successively, potentially obscuring the effects that each might have if applied individually to the raw set of sites. A detailed description of all filters is given in the methods section of the main text.

For each of four trios within the larger pedigree, we examined ∼1.04 Gb of sequence for DNMs after filtering for coverage and sequencing and alignment quality. This number of bases represents ∼43.5% of the 2.39 Gb of sequence in the dog reference genome (fig. 2). The majority of sequence removed by our filtering was due to sites containing repetitive DNA (identified using the dog-specific repeat library) and due to sites where three or more mapped reads contained gaps in their alignment to the reference genome. The overall number of sites remaining after filtering did not differ substantially among trios (fig. 2).

**Fig. 3.**
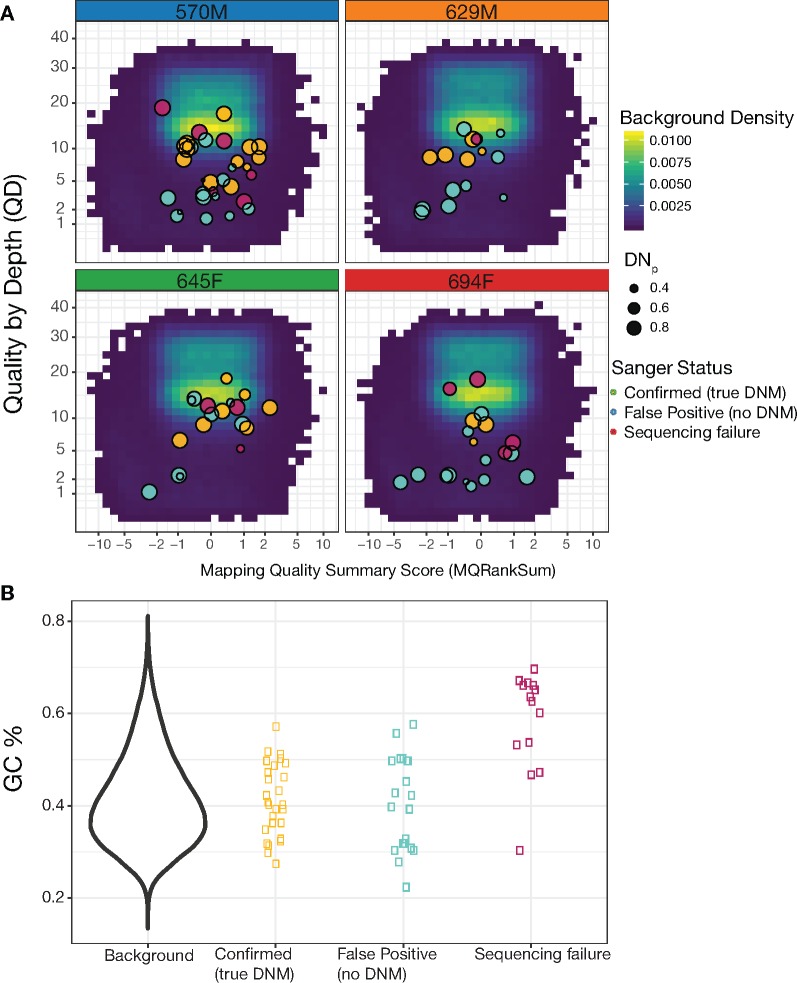
Sanger sequencing of potential DNMs. (*A*) Two different quality scores at sites chosen for Sanger sequencing are plotted against the background distribution of scores from sites within the same trio that passed all filters and had at least one alternative read. MQRankSum is a measure of mapping quality, where negative values indicate that reads with alternative alleles mapped less well than reads with reference alleles, potentially reflecting mismapped reads. QD measures base quality score normalized by sequencing depth and is a metric for sequencing quality. (*B*) GC content in 100 bp surrounding sites chosen for Sanger sequencing is compared with the GC content in the rest of the genome that passed all filters. Sites where Sanger sequencing failed often fell in regions with high GC content, motivating additional filtering based on GC content.

For each trio, we considered a site a potential DNM if the offspring had at least one alternative read from the reads observed in the parents (table 1). For each potential DNM site, we used genotype likelihoods output by GATK to calculate a de novo score (DN_p_, eq. 1) that can be roughly interpreted as the probability of containing a DNM (Ramu et al. 2013). We then applied a cutoff of DN_p_ > 0.3, which we chose to balance the probability of false negatives with the number of sites requiring manual examination of alignments and Sanger verification. Offspring 570M had fewer sites with at least one alternative read and apparent homozygous parents than the other offspring. An approximately equal number of these passed the DN_p_ cutoff in 570M and approximately twice as many passed the manual inspection when compared with the other offspring (table 1). In total, 84 sites were chosen for Sanger sequencing, 22 of which were chosen using a preliminary version of the pipeline (see supplementary table S1, Supplementary Material online, for more details). All true DNMs in the preliminary set were also present in the final set. We obtained Sanger sequencing results for 70 of the 83 sites, and of these 70 sites, 27 (38.6%) were confirmed as DNMs, and 43 (61.4%) were false positives. Of the confirmed DNMs, 12, 5, 7, and 3 were found in the offspring 570M, 629M, 645F, and 694F, respectively (table 1). We compared the sequencing and mapping quality of putative DNMs, using the QualByDepth (QD) and MappingQualityRankSumTest (MQRankSum) scores output by GATK, to the background distribution from sites passing all filters and where at least one alternative read was observed in each trio. We found that sites with low quality scores (QD < 4) tended to be false positives, as were those with low mapping qualities for reads with alternative alleles (MQRankSum < −2, fig. 3*a*). Among potential DNMs with quality scores within the typical range, there were still many false positives (fig. 3). Sites that failed Sanger sequencing showed a similar distribution of quality scores as the true DNMs.

**Table 1.** Examination of Potential DNMs.

	YNP 570M	YNP 629M	YNP 645F	YNP 694F
≥1 alt. read	2,676	3,529	3,935	3,225
DN_P_ > 0.3	112	109	106	108
Sanger sequenced	32	15	18	19
Failed	6	1	3	4
Confirmed DNM	12	5	7	3

Note.—The number of sites in each trio after all filtering steps, having a DN_p_ score >0.3, and chosen for Sanger sequencing. The final two rows give the number of confirmed DNMs and the number that failed to sequence out of those for which Sanger sequencing was attempted.

A likely reason for the Sanger sequencing failure at 14 of the potential DNM sites is high GC content within the region to be amplified. Nine out of the 14 failures had a GC content >60% within the 100 bp surrounding the site of interest (fig. 3*b*). In contrast, none of the sites where sequencing succeeded had a GC content this high. We therefore applied an additional filter removing all sites with >60% GC content within 100 bp. This filter removed another 5% of sites overall. We present mutation rate estimates both with and without this filter for high GC content.

The locations of the 27 DNMs validated using Sanger sequencing largely matched the genomic proportion of sequence in protein-coding, intronic, and intergenic regions observed in the reference genome and in variants that were transmitted from parents to offspring after filtering (supplementary fig. S8, Supplementary Material online). The nucleotide substitutions of validated DNMs also matched those of transmitted variants with the exception of an overabundance of A to T transversions (supplementary fig. S9, Supplementary Material online). Although the nucleotide context surrounding a site is known to have a large impact on local mutation rates (Aggarwala and Voight 2016), the number of DNMs discovered here is underpowered to detect such effects. Additionally, we observed a statistically significant excess of DNMs on chromosome 10 (*P* = 0.004, multinomial test) and in subtelomeric regions, defined as 5 Mb from the ends of assembled chromosomes (Webber and Ponting 2005) (*P* = 0.012, binomial test), compared with segregating variants that were transmitted from parents to offspring (supplementary figs. S10 and S11 and supplementary table S1, Supplementary Material online). Four of the five DNMs observed on chromosome 10 were found in offspring 570M and represent two pairs of DNMs with intermutation distances of about 40 and 70 kb.

### DNM Rate Estimation

**Fig. 4.**
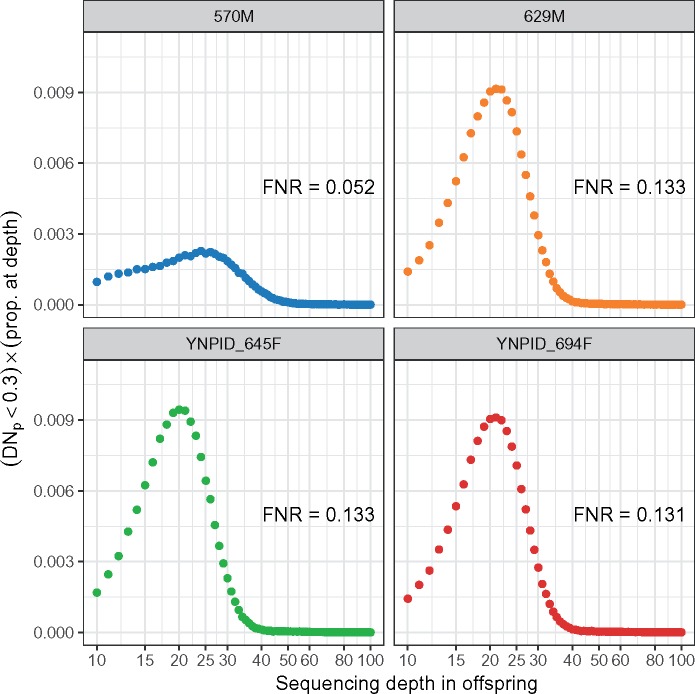
Trio false negative rates (FNRs) by sequencing depth in the offspring. FNRs were estimated for each possible sequencing depth in each offspring, then were multiplied by the fraction of sites in the offspring with that depth of coverage. This provides the contribution from each sequencing depth to the overall FNR at sites passing all filters. The overall FNRs are the sum of these points.

In order to estimate the mutation rate, it was necessary to estimate the FNR for each trio. This was done by creating synthetic DNMs by randomizing genotype likelihoods at sites passing all filters and calculating the proportion with DN_p_ < 0.3. The trio containing individual 570M had a lower FNR than the trios containing his half-siblings (fig. 4 and supplementary fig. S2, Supplementary Material online). This lower rate probably reflects higher genome coverage of this individual and his father, 302M. In general, the fraction of simulated DNMs with DN_p_ < 0.3 does not decrease to 0 as the sequencing depth of an offspring increases (supplementary fig. S11, Supplementary Material online). This finding likely reflects lower coverage in some parents and missed parental heterozygotes. Interestingly, the FNR actually increases for higher sequencing depths, perhaps because higher depths are enriched for mismapped reads that appear as infrequent alternative alleles within the reads of offspring. Infrequent alleles among reads are likely to generate lower DN_p_ scores because they can be modeled as sequencing errors. However, because the fraction of sites with high-enough read depths to elevate the FNR is low, such sites do not contribute much to the overall FNR (fig. 4). Finally, filtering for high GC content had almost no impact on the estimated FNRs (supplementary fig. S3, Supplementary Material online).

Given that some potential DNM sites failed to produce Sanger sequencing data, we provide broad bounds on the per-generation mutation rate by considering the cases where none of the failed sites are DNMs and cases where all of them are DNMs. We calculate the posterior distributions of the mutation rate for both cases and provide the 5th percentile of the distribution for the minimum number of mutations and the 95th percentile of the distribution for the maximum number of mutations. This procedure is done with and without the additional filter for high GC content regions.

**Fig. 5.**
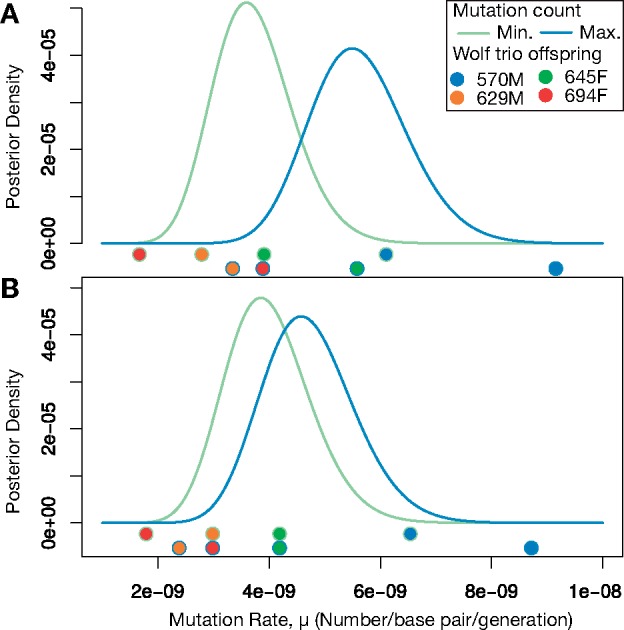
Posterior distributions on the mutation rate. (*A*) Posterior distributions on the per-generation mutation rate without filtering based on GC content. The curve corresponding to the minimum mutation count is the posterior distribution we would calculate if all candidate sites that failed Sanger sequencing are false positives. The curve corresponding to the maximum mutation count is the posterior distribution we would calculate if all failed candidate sites are true DNMs. Dots beneath the curves show the point estimates of the mutation rates that would be obtained from the different trios. (*B*) Posterior distributions on the per-generation mutation rate after filtering based on GC content.

Estimated mutation rates with and without filtering high GC content regions are largely concordant. The posterior distributions of the mutation rate without filtering for GC content bound the mutation rate within the range (2.6 × 10^−9^, 7.1 × 10^−9^) (fig. 5*a*). Filtering regions with high GC content results in a narrower bound for the mutation rate of (2.8 × 10^−9^, 6.2 × 10^−9^) (fig. 5*b*) by contracting the posterior distributions for the minimum and maximum number of mutations and also moving them closer together. This second range is nested within the first, and the average posterior mean mutation per generation per base pair rate across GC filtering and minimum and maximum numbers of mutations gives a point estimate of ∼4.5 × 10^−9^.

## Discussion

Mutation rates are necessary to scale times in population genetic models from units of mutations to units of generations. When combined with a generation time, model parameters can then be scaled in units of years. Given that the divergence time between dog and wolf populations provides an upper bound on the timing of dog domestication, it is necessary to scale population genetic models of canine history using the correct mutation rate. Although previous estimates of the mutation rate are available based on fossil-calibrated mammal phylogenies (Kumar and Subramanian 2002) and on ancient DNA from wolves and dogs (Skoglund et al. 2015; Frantz et al. 2016), we provide the first direct estimate based on sequencing parents and offspring. We bound the mutation rate in wolves to 2.6–7.1 × 10^−9^ per base pair per generation with a point estimate of 4.5 × 10^−9^.

The filters we used to remove regions of the genome enriched for false positives could potentially bias our estimated mutation rate. For instance, we removed regions of the genome with a large number of variants. Areas with high genetic diversity may be regions of the genome with elevated mutation rates, or, alternatively, heterozygosity itself may have an impact on the mutation rate (Yang et al. 2015). However, because the fraction of the genome removed by the high-variant filter was small (∼4%), the elevation of the mutation rate in these regions would have to be large to have a meaningful bias. The mutation rate in highly variable regions would need to be about 26 times greater than the mutation rate in the rest of the genome in order to double it. A large portion of filtered sites were removed due to occurrence in repetitive regions (fig. 2). Given that studies of human mutations have measured higher mutation rates in ancient repeats than in nonrepetitive regions (Turner et al. 2017), we acknowledge that the mutation rate calculated in the present study may be an underestimate. In general, when our estimated mutation rate is used in future population genetic studies, researchers should use similar filtering strategies to avoid downstream biases in their analysis.

Crucially, our bounds on the mutation rate exclude the value 1.0 × 10^−8^ which had been used by studies prior to the estimates from ancient DNA (Lindblad-Toh et al. 2005; Freedman et al. 2014; Skoglund et al. 2015). Our point estimate aligns closely with rates estimated using ancient DNA. Skoglund et al. (2015) and Frantz et al. (2016) estimated rates of 4.0 × 10^−9^ and 3.0–4.5 × 10^−9^ per generation, respectively. Two assumptions of the methodology used in these studies can bias mutation rate estimates. First, radiocarbon dates of ancient samples will be underestimates of divergence times between the ancestral populations of ancient and modern individuals and will bias mutation rates upward. Second, assuming a lack of postdivergence gene flow will bias mutation rates downward. Given the agreement of our estimate with those from ancient DNA studies, it may be that these biases are small or that their opposing directions tend to cancel out. However, we are unable to rule out a mutation rate ∼50% lower (2.6 × 10^−9^) or higher (7.1 × 10^−9^) than 4.5 × 10^−9^. We urge caution in accepting a simple point estimate on the grounds that it coincides with estimates based on ancient DNA. The method used by Skoglund et al. (2015) and Frantz et al. (2016) does not account for uncertainty in the inferred demographic history that would propagate into mutation rate estimates, and relies on dated remains for the divergence time between ancestral populations. More work is needed to characterize the bias and variance of mutation rate estimates generated with that method.

To get a sense for the uncertainty remaining in population genetic models of canid demographic history, we rescaled the estimated times of several important events taken from previously published models (table 2). Because previous studies used point estimates of the mutation rate to calibrate their analyses, our recalibration has the effect of increasing uncertainty in the timing of specific events. For instance, Frantz et al. (2016) estimated that the divergence between East Asian and Western Eurasian dogs occurred around 6,400–14,000 years ago. They used the fact that this postdates the oldest known dog remains in Europe around 15,000 years ago (Pionnier-Capitan et al. 2011) to argue that domestication occurred independently in Europe and Asia and that Western Eurasian dogs were largely replaced by those with East Asian origin. If the true mutation rate is on the lower end of our interval, the upper bound on the divergence time estimated by Frantz et al. (2016) is pushed back to ∼17,000 years ago. The divergence between East Asian and Western Eurasian dogs could therefore have occurred before the first appearance of dog remains in the European archeological record. If so, this would be consistent with a single domestication event.

**Table 2.** Recalibration of Estimated Divergence Times in Canid History.

Divergence Event	Published Dates (ka)	Our Recalibration (ka)
Western Eurasian dogs | East Asian dogs	LF: 6 (6–11)	5 (4–17)
Mexican wolves | Yellowstone wolves	BvH: 14 (12–18)	12 (8–28)
	ZF: 14 (10–17)	12 (7–26)
Basenji | other dogs	AF: 32 (29–34)	28 (19–52)
	ZF: 21 (19–23)	19 (12–35)
European wolves | East Asian wolves	AF: 33 (29–38)	29 (19–58)
	BvH: 27 (24–30)	24 (16–46)
Dogs | wolves	AF: 37 (35–40)	33 (23–62)
	BvH: 28 (24–30)	25 (16–46)
	ZF: 29 (24–30)	26 (16–46)
	LF: 34 (17–48)	30 (11–74)
North American wolves | Eurasian wolves	BvH: 31 (28–32)	28 (18–49)
	ZF: 31 (29–33)	28 (19–51)
Coyotes | wolves	BvH: 165 (158–171)	146 (102–264)
Golden Jackals | Coyote/Wolf ancestors	AF: 995 (797–1,038)	884 (514–1,596)

Note.—Estimated divergence times were taken from four studies that used coalescent models to reconstruct canid population history. Times from different papers have been denoted as follows: AF (Freedman et al. 2014), BvH (vonHoldt et al. 2016), ZF (Fan et al. 2016), and LF (Frantz et al. 2016). The AF, BvH, and ZF studies used the Generalized Phylogenetic Coalescent Sampler method (G-PhoCS) (Gronau et al. 2011). Point estimates represent posterior means and intervals are 95% credible intervals. The LF study used relative cross-coalescent rates calculated using MSMC (Schiffels and Durbin 2014) to estimate divergence times. Point estimates are the times when between population coalescent rates exceeded 50% of the within-population coalescent rates, and intervals give the corresponding times for 25% and 50% of the within-population coalescent rate. Published dates from Freedman et al. (2014) were scaled to a mutation rate of 4.0 × 10^−9^ to be comparable with other studies. We recalibrated divergence times by rescaling point estimates using our estimated mutation rate of 4.5 × 10^−9^. Lower bounds on divergence times were obtained by rescaling the lower bound on estimated rates using our upper bound on the mutation rate, 6.2 × 10^−9^, and upper bounds on divergence times were obtained by rescaling the upper bound on estimated rates using our lower bound on the mutation rate, 2.8 × 10^−9^.

Our recalibration suggests a wide range of possible dates for the divergence of the ancestral populations of dogs and Eurasian wolves. Recalibrated point estimates from four studies range from 25,000 to 33,000 years ago. However, the interval reported by Frantz et al. (2016) based on cross-coalescent rates calculated using multiple sequentially Markovian coalescent (MSMC) is recalibrated to 11,000–74,000 years ago, whereas intervals based on G-PhoCS models range from 16,000 to 64,000 years ago (table 2). These time intervals span the range of the first appearance of dogs in the fossil record to around the time when anatomically modern humans left Africa. Thus, although the most likely period for the divergence of dog and wolf ancestors is between 19,000 and 33,000 years ago, we cannot yet fully rule out earlier or later divergence dates, and a greater timespan for dog domestication is therefore also possible.

The use of accurate generation times is of equal importance as accurate mutation rates during calibration of population genetic models. On phylogenetic time scales, changes in the generation time and other life-history traits are known to affect substitution rates (Wu and Li 1985; Sayres et al. 2011; Moorjani et al. 2016). It has been suggested that the commonly used generation time of 3 years in canid genetics might be too low since generation times between 3 and 5 years have been estimated in contemporary populations (vonHoldt et al. 2008; Stahler et al. 2013; Mech et al. 2016; Mech and Barber-Meyer 2017). Changes in the generation time associated with dog domestication would also lead to changes in the per-year mutation rate and therefore bias estimates of divergence times when scaling genealogies by our estimated mutation rate. We note that, if a generation time other than 3 years is desired, the generation times will linearly scale all the estimates given in table 2.

One surprising result of the work presented here, and possible reason for caution, is the ∼2-fold greater number of mutations observed in 570M relative to the offspring of 480M. 570M did not have a greater amount of sequence passing filters than his half-siblings (fig. 2). Additionally, although the estimated FNR was lower for 570M, we would only expect this to increase the number of mutations found by about 10%. Conceivably, the overall FNR in the other offspring may have been underestimated, but the rate would have to be ∼50% to explain the observed number of mutations.

Another possible explanation for the greater number of DNMs observed in 570M is due to this wolf having an older father (302M). More mutations accumulate with paternal age in primates (Kong et al. 2012; Venn et al. 2014; Rahbari et al. 2016; Thomas et al. 2018). The paternal age at birth of 570M was 6 years, and the paternal ages of 629M, 645F, and 694F were 3, 4, and 3 years, respectively (supplementary fig. S4, Supplementary Material online). The 2-fold greater number of mutations observed in 570M is therefore consistent with a paternal age effect, as is the higher mutation count in 645F relative to her siblings (table 1). However, we caution against overinterpretation of these observations given our extremely limited sample size. In humans ∼40 years of the father’s life are needed to double the mutation rate (Kong et al. 2012; Rahbari et al. 2016), although the rate of increase observed in chimpanzees and owl monkeys is faster (Venn et al. 2014; Thomas et al. 2018). The paternal age effect thus remains a potential explanation for the differences in DNMs among the wolves studied here. More sequencing on wolves with greater variation in paternal age would be necessary to show whether age has a substantial effect. Our pedigree contains even less variation in maternal age at birth: 570M, 629M, and 694M were from the same litter when 569F was 2 years old, and 645F was born the next year. Two and three are on the low end for observed maternal ages in North American wolves (vonHoldt et al. 2008; Stahler et al. 2013; Mech et al. 2016; Mech and Barber-Meyer 2017). If there is a strong maternal age effect in wolves, this could bias our mutation rate estimate downward.

We also observe statistically significant clustering of DNMs in nucleotide substitutions, chromosomes, and subtelomeric versus interstitial DNA when compared with the genomic background of observable sites and to variant sites transmitted from parents to offspring (supplementary figs. S9–S11, Supplementary Material online). Although we lack the sample size to investigate these patterns in greater detail, we note that certain processes generate spatially clustered mutations (Chan and Gordenin 2015) and that mutation rate is positively correlated with recombination rate in humans (Kessler et al. 2019), whereas recombination rate is positively negatively correlated with distance to telomeres in dogs (Campbell et al. 2016).

Variation in the mutation rate may exist among individuals, as has been observed among human trios (Conrad et al. 2011; Kessler et al. 2019). Consequently, the mutation rate estimated here may not accurately represent gray wolves in general given that only three parents from one population were sampled. Finally, some number of the excess DNMs identified in 570M may be somatic mutations. Without assessing if any of these mutations were transmitted to a future generation, we cannot be sure that the mutations identified here occurred in the parents’ germ line. Multigenerational sequencing approaches, such as has been implemented in African green monkeys (Pfeifer 2017a), would resolve this issue and should be preferred when feasible.

The evolution of mutation rates is another arena in which pedigree-based estimates of mutation rates such as ours are useful. In particular, the drift-barrier hypothesis for the evolution of mutation rates predicts a negative correlation between mutation rate and effective population size (Sung et al. 2012; Lynch et al. 2016). Such a negative relationship is observed within the eubacteria, unicellular eukaryotes, and multicellular eukaryotes for which mutation rates have been measured (Lynch et al. 2016; Smeds et al. 2016; Pfeifer 2017a). Our mutation rate estimate therefore also represents a step toward understanding the evolution of mutation rates, especially in wild species who have close common ancestry with domestic forms.

Aside from mice (Uchimura et al. 2015), ours is also the only directly estimated mutation rate in a nonprimate mammal. In nonmodel species that cannot be readily bred in captivity, or that have long generation times, pedigree sequencing will remain the only way to directly estimate mutation rates. An unfortunate step in this process, as it currently exists, is the need for a manual examination of alignments at putative DNMs and resequencing. Filtering is necessary to limit the number of alignment plots that must be examined. The future development of computational methods that eliminate the need for manual inspection would make it much easier to estimate mutation rates from a large number of trios, especially in nonmodel organisms lacking highly curated repeat libraries.

## Materials and Methods

### Sampling Strategy and Whole-Genome Sequencing

We sequenced whole genomes for a known pedigree of seven wolves (vonHoldt et al. 2008; Schweizer et al. 2018) containing one mother and her four offspring from two different fathers (fig. 1). Samples from 569F and 570M were sequenced as part of Fan et al. (2016; NCBI SRP044399) using the HiSeq 2000 platform and are described in that study as the “Yellowstone trio.” The prior sequencing of 302M suffered from 50% PCR duplicates, so we sequenced an additional four lanes of the Illumina HiSeq 2500 platform using a different library preparation, as described below. The other four individuals were sequenced on one lane each using the Illumina HiSeq 4000 platform. All libraries were sequenced with a 100-bp paired-end strategy. Libraries for 302M and the four new individuals (480M, 629F, 645F, and 694M) were prepared as part of a previous study (Schweizer et al. 2018). Briefly, genomic DNA was sheared to ∼300–450 bp using a Biorupter NGS Sonication System, then libraries were prepared using a “with-bead” protocol (Faircloth 2015) and unique 6-bp indexes (Faircloth and Glenn 2012). We note that, although Schweizer et al. (2018) sequenced targeted enrichment libraries, for the present study we sequenced the original precapture libraries to obtain whole-genome data.

### Calculation of Genotype Likelihoods

To identify DNMs with a low FNR, we employed a strategy of liberally calling potential DNMs and then detecting false positives for exclusion via independent sequencing. Sequencing data from all seven individuals were passed through a series of processing and filtering steps to generate a set of putative DNMs that were subsequently tested by Sanger sequencing (supplementary fig. S1, Supplementary Material online). We aligned sequencing reads to the dog reference genome version CanFam3.1 using BWA 0.7.12 (Li and Durbin 2009). Although a wolf reference genome exists, it is not as complete as the dog genome and using this genome does not substantially impact analyses when compared with CanFam3.1 (Gopalakrishnan et al. 2017). We used GATK 3.5.0 (DePristo et al. 2011) to realign alignments around indels and remove duplicates. To generate an initial trial set of variants for recalibrating base quality scores, we used GATK’s UnifiedGenotyper to call variant sites, and kept SNP sites if they passed the recommended hard filtering thresholds (QD > 2, FS < 60, MQ > 40, MQRankSum > −12.5, and ReadPosRankSum > 15). We filtered sites in repetitive regions from this set using RepeatMasker 4.0.6 and a dog-specific repeat library (Smit et al. 2013–2015), then treated the remaining set of variants as “known” to recalibrate base quality scores using GATK. After recalibrating base quality scores, we used the GATK UnifiedGenotyper algorithm to calculate genotype likelihoods at all sites with a minimum base quality score of 15 and the “emit all sites” options. Genotype likelihoods calculated in this manner are independent for each individual. We retained all sites regardless of their variant quality scores in order to avoid bias against variable sites and therefore potential DNMs.

### Site Filters

We chose to apply site-level filters per trio so as to maximize the likelihood of observing true DNMs in each trio. We selected our filters to remove genomic regions likely to be enriched for false positives, to retain sites with sufficient coverage and sequencing quality. As with the variant set used in recalibration, we first filtered sites in repetitive regions using RepeatMasker (Smit et al. 2013–2015). We then removed sites marked by GATK as missing, either as a result of a sequencing depth of zero or if more than 5% of reads spanning the locus contain deletions. We also filtered sites with <10-fold or >100-fold coverage in any individual because low coverage sites have low genotype quality and high coverage sites may have many mismapped reads due to copy number variation (Keightley et al. 2014; Pfeifer 2017b). In order to only examine sites where the parents were confidently homozygous for the same allele, we removed sites with one or more alternative alleles observed in the parents’ mapped reads. We applied two additional filters to account for base quality and mismapped reads. First, we removed sites with four or more variant sites within a 200-base window on either side in order to capture the approximate range for which primers were designed, and, second, we removed sites where three or more of the reads mapping to that site contained gaps in their alignments. We consider potential biases introduced by these filters on the final estimates in the Discussion section.

### Identification of DNMs

The above procedure yields a set of sites for each trio in the family where the parental individuals appear homozygous for the same allele. Although the vast majority of these sites are homozygous in the offspring as well, a small number contain DNMs. To find these mutations, we begin with the list of all sites where one or more alternative alleles are observed in the offspring. Other studies have found that, among sites with at least one alternative read in the offspring and no alternative read in the parents, the vast majority are sequencing errors, missed heterozygous genotypes in the parents, or mismapped from elsewhere in the genome (Keightley et al. 2014, 2015; Smeds et al. 2016; Pfeifer 2017b). To distinguish sites with true DNMs from those with sequencing errors or missed parental heterozygotes, we calculated a de novo score (DN_p_) that can be roughly interpreted as the probability that each site contains DNM. This calculation was made by considering the posterior probability of each genotype combination between parents and offspring (Ramu et al. 2013) and computing the probability of configurations that require a DNM event:

(1) $$P(G_{C},G_{M},G_{F}|D)\propto \frac{\overset{\mathrm{genotype}\mathrm{likelihoods}\mathrm{of}\mathrm{observed}\mathrm{individuals}}{\overset{︷}{P(D_{M}|G_{M})P(D_{F}|G_{F})P(D_{C}|G_{C})}}}{\times \underset{\mathrm{transmission}\mathrm{probability}}{\underset{︸}{P(G_{C}|G_{M},G_{F})}}\underset{\mathrm{parental}\mathrm{heterozygosity}}{\underset{︸}{P(G_{M},G_{F}|\theta )}}}.$$

The term for transmitting different genotypes to the offspring contains an assumed mutation rate and the term for the parental heterozygosity contains a parameter for the heterozygosity in the population (Ramu et al. 2013). The purpose of this calculation is to weigh evidence from genotype likelihoods, which take into account both sequencing depth and quality, with our prior belief about how often mutations occur and how likely the parents are to be homozygous at a given site. Given the fact that base qualities may be a poor reflection of the actual probability of sequencing errors, we do not interpret the de novo scores calculated using this formula as true probabilities but rather as scores with which to rank potential DNMs. We first calculated DN_p_ using a mutation rate of 4 × 10^−9^ per generation, as estimated by Skoglund et al. (2015), and a heterozygosity of 0.0015 as values close to this have been observed in many wolf populations (Freedman et al. 2014; Fan et al. 2016; Schweizer et al. 2018). These realistic parameters yielded very few candidate DNMs and a large number of clear false positives, potentially because base quality score recalibration was overly conservative. As part of our strategy to minimize the FNR by calling DNMs more liberally, we calculated DN_p_ with the mutation rate prior set to 1.0 × 10^−6^ per generation. The heterozygosity was set to 0.008 to try and avoid missed parental heterozygotes. Only the genotype combination where the offspring is heterozygous and both parents are homozygous for the reference allele was considered compatible with a DNM. Sites with a DN_p_ > 0.3 were examined further.

Among sites passing all filters and having a DN_p_ > 0.3, many contained, on visual inspection, obvious sequencing errors or had a high proportion of mismapped reads. This is a problem faced by many studies that attempt to estimate the mutation rate using pedigree sequencing (Keightley et al. 2014, 2015; Smeds et al. 2016; Pfeifer 2017a). Following the example set by Keightley et al. (2014), we removed these sites from further analysis by examining read alignments manually using IGV 2.3.79 (Robinson et al. 2011; Thorvaldsdóttir et al. 2013) and the *igv_plotter* library (Weisburd 2017). IGV plots often clearly showed, in the form of high numbers of mismatches and gaps in the alignment, whether a site was in a region where reads tended to mismap (see examples in supplementary figs. S5–S7, Supplementary Material online). In addition to visualizing alignments, we used the QD and MQRankSum quality metrics output by GATK of potential DNMs to compare them to other variants in the sample. QD reflects the depth-normalized sequencing quality and MQRankSum reflects how well alternative versus reference reads map. If a putative DNM had QD and MQRankSum scores within the typical range of other variants in the sample and in addition having clean alignments when visually inspected, that site was selected for validation by Sanger sequencing. Some sites outside the typical background range of QD and MQRankSum were sequenced as well to check that our approach was not generating false negatives.

As stated above, we used relaxed parameters to choose sites for validation in order to minimize the number of false negatives. To confirm our interpretation of alignment plots, we also Sanger sequenced some sites that, from inspection of alignment plots, appeared to be clear examples of mismapping or sequencing errors. The pipeline described above was implemented using Snakemake (Köster and Rahmann 2012) and is available on github (https://github.com/emkoch/wolf-dnm-pipeline; last accessed July 8, 2019).

### Mutation Rate Calculation

In order to calculate an estimate of the mutation rate given a set of verified DNMs, it is necessary to know how many mutations could have potentially been observed from each trio. We calculated this number by taking the number of sites in each trio that passed all filters and multiplied it by one minus an estimated FNR for that trio (see below). For computational reasons, it was impractical to apply the filter for gaps in read alignment to the whole genome, so we estimated the proportion of sites removed by applying the filter to a random sample of sites.

The FNR for each trio is the probability that a site containing a true DNM and passing all filters would have a DN_p_ < 0.3. To approximate this probability, we used an assumption that, conditional on a true DNM having a DN_P_ > 0.3, it would be chosen for validation by independent sequencing after having its alignment examined, and that sequencing would indicate a DNM event. That is, we assume that no true DNM which passed the DN_P_ cutoff would have been discarded because its alignment resembled a sequencing or alignment error. Under this assumption, we are able to calculate the FNR by generating a set of simulated DNMs from the genotype likelihoods calculated in each trio, calculating the DN_p_ for each simulated DNM, and measuring the proportion which fell below 0.3. A simulated DNM was created by first sampling genotype likelihoods from a site where both the offspring and at least one of the parents were heterozygous. These likelihoods were then paired with genotype likelihoods for the parents chosen at random from the set of sites where no alternative alleles were observed. DN_p_ values were calculated using equation (1) just as for the real data. This approach also assumes that the joint coverage distribution at real DNMs would be the same as that for the randomized, simulated set.

Using the number of sites passing all filters and an estimate of the FNR, we calculated a posterior distribution on the per-generation mutation rate using

$$\begin{array}{c} \sum_{i}X_{i}∼\;\mathrm{Poisson}\left(\sum_{i}2L_{i}(1-\beta_{i})\mu \right)\\ p(\mu )\propto \sqrt{\frac{1}{\mu }}, \end{array}$$

where *X_i_* is the total number of DNM we observed in trio *i*, *L_i_* is the number of sites passing all filters in trio *i*, and *β_i_* is the estimated FNR for trio *i*. *μ* denotes the mutation rate per generation. We used a prior on *μ* proportional to the inverse square root. This is the Jeffrey’s prior (an uninformative prior that is invariant to reparameterization) for a Poisson rate parameter.

## Supplementary Material

Supplementary data are available at *Molecular Biology and Evolution* online.

## Supplementary Material

msz159_Supplementary_Data
