# Supplementary material for: De Novo Mutation Rate Estimation in Wolves of Known Pedigree
Source: Mol Biol Evol. 2019 Jul 12;36(11):2536–47. doi: 10.1093/molbev/msz159 (PMC6805234; doi:10.1093/molbev/msz159)
Supplement: msz159_Supplementary_Data [file msz159_supplementary_data.zip › dnm_supplemental_figure_captions.pdf]

Fig. S1. **Bioinformatics pipeline.** The basic workflow of our bioinformatics pipeline, including alignment processing, site filtering, identifying candidate mutations, determining which sites should be validated, and use of Sanger sequencing to identify a verified set of candidate *de novo* mutations. Genotype likelihoods were calculated after alignment processing and before site filters were applied. See text for more details of methods.

Fig. S2. **Full distributions of sequencing depths in each in individual in the pedigree.** Sequencing depths were calculated on processed alignment files and do not reflect any subsequent filters.

Fig. S3. **Trio false negative rates by sequencing depth in the offspring after filtering for high GC content.** The same procedure for estimating false negative rates was performed after filtering for high GC content. False negative rates were estimated for each possible sequencing depth in each offspring and these were multiplied by the fraction of sites in the offspring with that depth of coverage. This provides the contribution from each sequencing depth to the overall false negative rate at sites passing all filters. The overall false negative rates are the sum of these points and do not differ substantially from those shown in Figure 5.

Fig. S4. **Birth year and paternal and maternal ages of wolves in the pedigree.** Points indicate the birth year of wolves in the pedigree. Exact dates of birth are not known, but litters are born in April of each year. For each offspring, the age of its mother and father at birth is provided.

Fig. S5. **Example alignment plot for a site with a large number of nearby sequencing errors.** Site 15458730 on chromosome 1 in the trio of individual YNPID 629. Variation at theis stie appears to be due to sequencing errors because that site exists at the end of a string of Ts and other C to T differences exist in the region.

Fig. S6. **Example alignment plot for a site with mismapped reads.** Site 40892472 on chromosome 7 in the trio of individual YNPID 645. The fact that the A to T change always appears on reads with an A to T change two nucleotides away indicates that the reads containing

these bases originate from elsewhere in the genome where the sequence is identical except for these two substitutions.

Fig. S7. **Example alignment plot for a confirmed *de novo* mutation.** Site 45648332 on chromosome 2 in the trio of individual YNPID 629. Few nearby sites contain sequencing errors or read patterns indicative of mismapping. Sanger sequencing confirmed that this site contains a *de novo* mutation.

Fig. S8. **Functional categories of validated *de novo* mutations.** Validated *de novo* mutations were assigned to annotation categories using variant effect predictor (VEP) (McLaren et al., 2016). The percentages in each category were compared to those observed in the genomic background after filtering and in variable sites that were transmitted from parents to offspring. Coding sites include all those within the protein-coding parts of exons. Intron sites include all sites within introns including those involved in splicing. Other includes all sites within additional categories such as untranslated regions or noncoding genes. Intergenic sites include all remaining sites. As expected, transmitted variants were depleted from protein-coding and intronic sites compared to new mutations and the genomic background. Error bars are 95% confidence intervals calculated for multinomial proportions (Sison & Glaz 1995, Villacorta 2012).

Fig. S9. **Nucleotide substitutions of validated *de novo* mutations.** The proportions of different nucleotide substitutions at validated *de novo* mutations were compared to those of transmitted variants in the sample after all filters were applied. Mutation types at *de novo* mutations largely match those at variable sites except for an overabundance of T to A substitutions. Error bars are 95% confidence intervals calculated for multinomial proportions (Sison & Glaz 1995, Villacorta 2012).

Fig. S10. **Proportions of validated *de novo* mutations on different chromosomes.** The proportions of validated *de novo* mutations on different chromosomes were compared to those of transmitted variants in the sample after all filters were applied. Chromosomal locations largely match those of transmitted variants with more mutations being observed on the larger chromosomes. However, chromosome 10 has a statistically significant excess of *de novo*

mutations when compared to variable sites. Error bars are 95% confidence intervals calculated for multinomial proportions (Sison & Glaz 1995, Villacorta 2012).

Fig. S11. **Genomic locations of validated *de novo* mutations.** The genomic coordinates of validated *de novo* mutations are shown for each chromosome that had at least one mutation. Subtelomeric regions are approximated as 5Mb from the start and end of the assembled chromosomes and are shown in grey.

Fig. S12. **Absolute false negative rates by sequencing depth.** False negative rates were estimated for each possible sequencing depth in each offspring. Sites with higher sequencing depths had higher false negative rates but because the overall number of sites sequenced at high coverage was low the impact on the overall false negative rate was minor.
