# Supplementary material for: De Novo Mutation Rate Estimation in Wolves of Known Pedigree
Source: Mol Biol Evol. 2019 Jul 12;36(11):2536–47. doi: 10.1093/molbev/msz159 (PMC6805234; doi:10.1093/molbev/msz159)
Supplement: msz159_Supplementary_Data [file msz159_supplementary_data.zip › figure_s1.pdf]

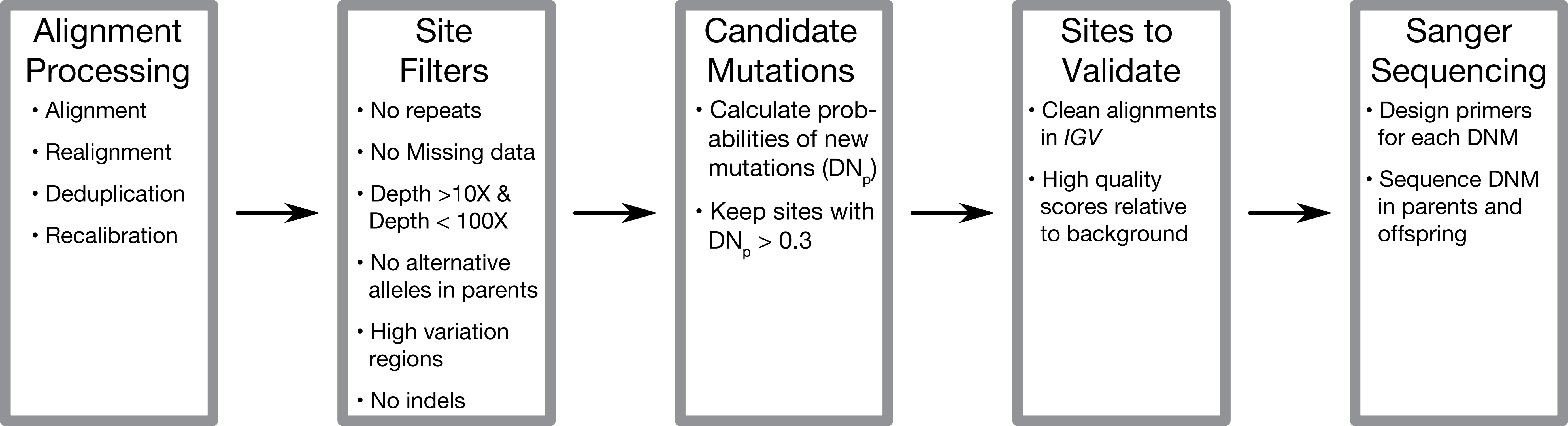
Alignment Processing
• Alignment
• Realignment
• Deduplication
• Recalibration
Site Filters
• No repeats
• No Missing data
• Depth >10X & Depth < 100X
• No alternative alleles in parents
• High variation regions
• No indels
Candidate Mutations
• Calculate probabilities of new mutations (DNp)
• Keep sites with DNp > 0.3
Sites to Validate
• Clean alignments in IGV
• High quality scores relative to background
Sanger Sequencing
• Design primers for each DNM
• Sequence DNM in parents and offspring
