# Supplementary material for: De Novo Mutation Rate Estimation in Wolves of Known Pedigree
Source: Mol Biol Evol. 2019 Jul 12;36(11):2536–47. doi: 10.1093/molbev/msz159 (PMC6805234; doi:10.1093/molbev/msz159)
Supplement: msz159_Supplementary_Data [file msz159_supplementary_data.zip › figure_s4_fixed.pdf]

YNP ID	Father's Age (years)	Mother's Age (years)	Birth Year 2000	2001	2002	2003	2004	2005	2006	2007
569F							○			
302M			□							
570M	6	2							□	
480M						□				
629M	3	2							□	
645F	3	3								○
694F	4	2							○	
