# Supplementary figures and images for: De Novo Mutation Rate Estimation in Wolves of Known Pedigree
Source: Mol Biol Evol. 2019 Jul 12;36(11):2536–47. doi: 10.1093/molbev/msz159 (PMC6805234; doi:10.1093/molbev/msz159)

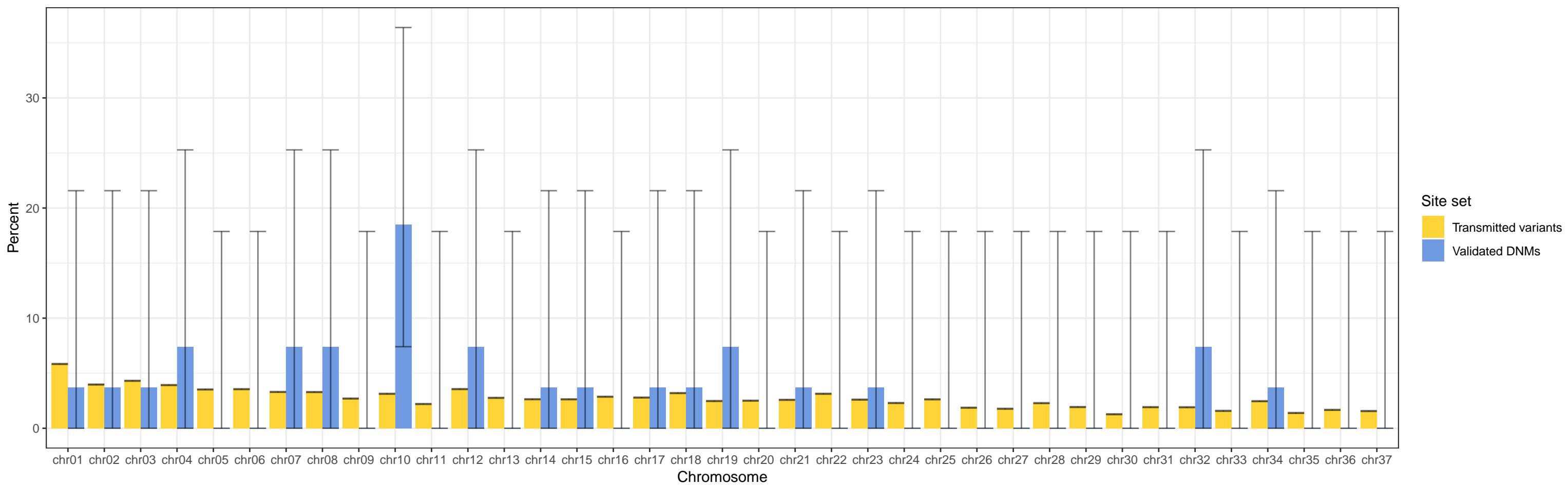

Supplement: msz159_Supplementary_Data [file msz159_supplementary_data.zip › figure_s10.pdf]

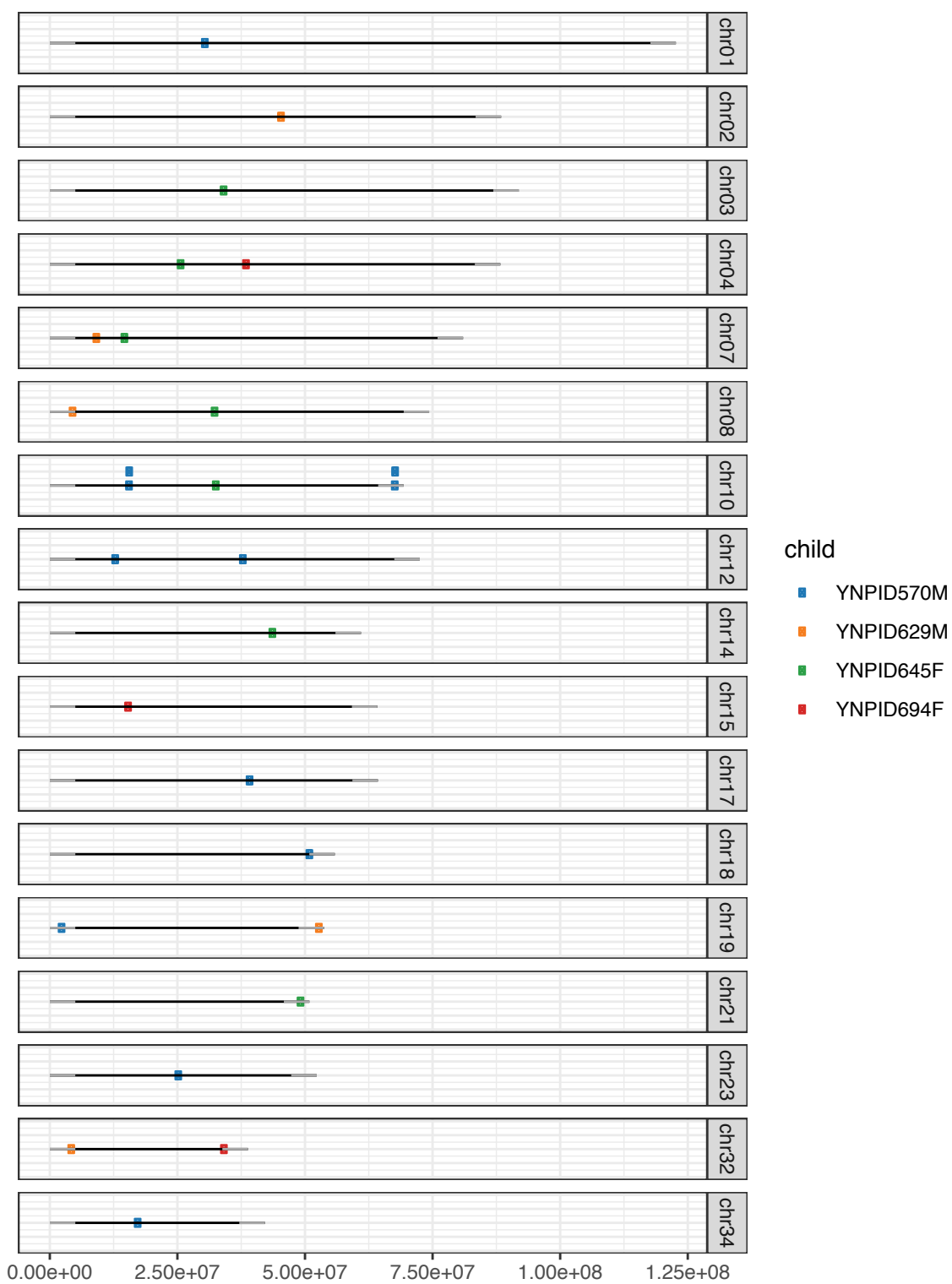

Supplement: msz159_Supplementary_Data [file msz159_supplementary_data.zip › figure_s11.pdf]

$DN_b < 0.3$

YNPID\_570M

0.4  
0.2  
0.0

YNPID\_629M

YNPID\_645F

0.4  
0.2  
0.0

YNPID\_694F

10 15 20 25 30 35 40 45 50 55 60 65 70 75 80 85 90 95 100

Sequencing depth in child

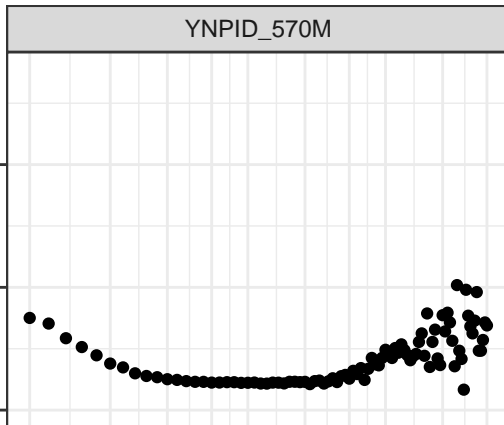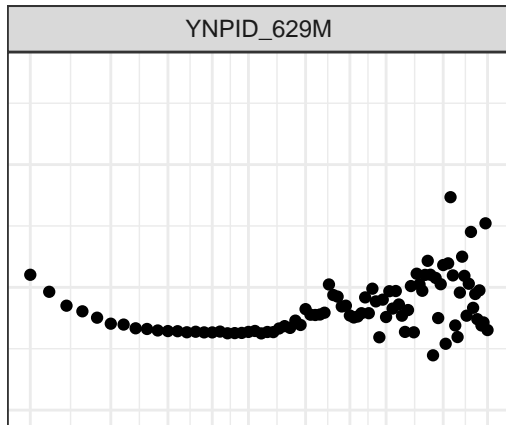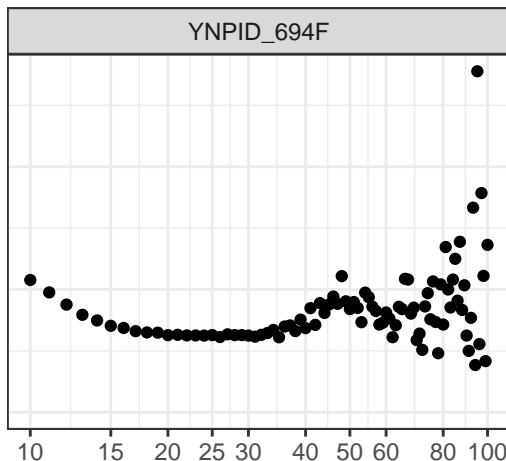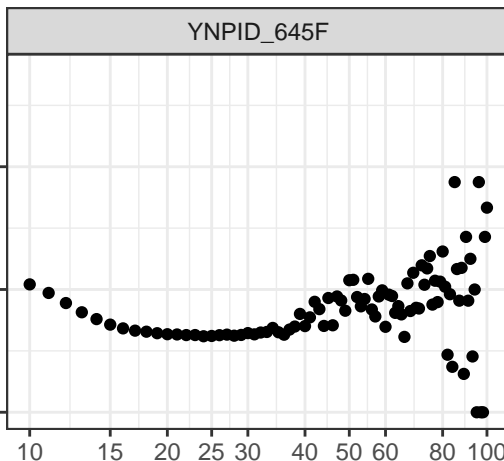

Supplement: msz159_Supplementary_Data [file msz159_supplementary_data.zip › figure_s12.pdf]

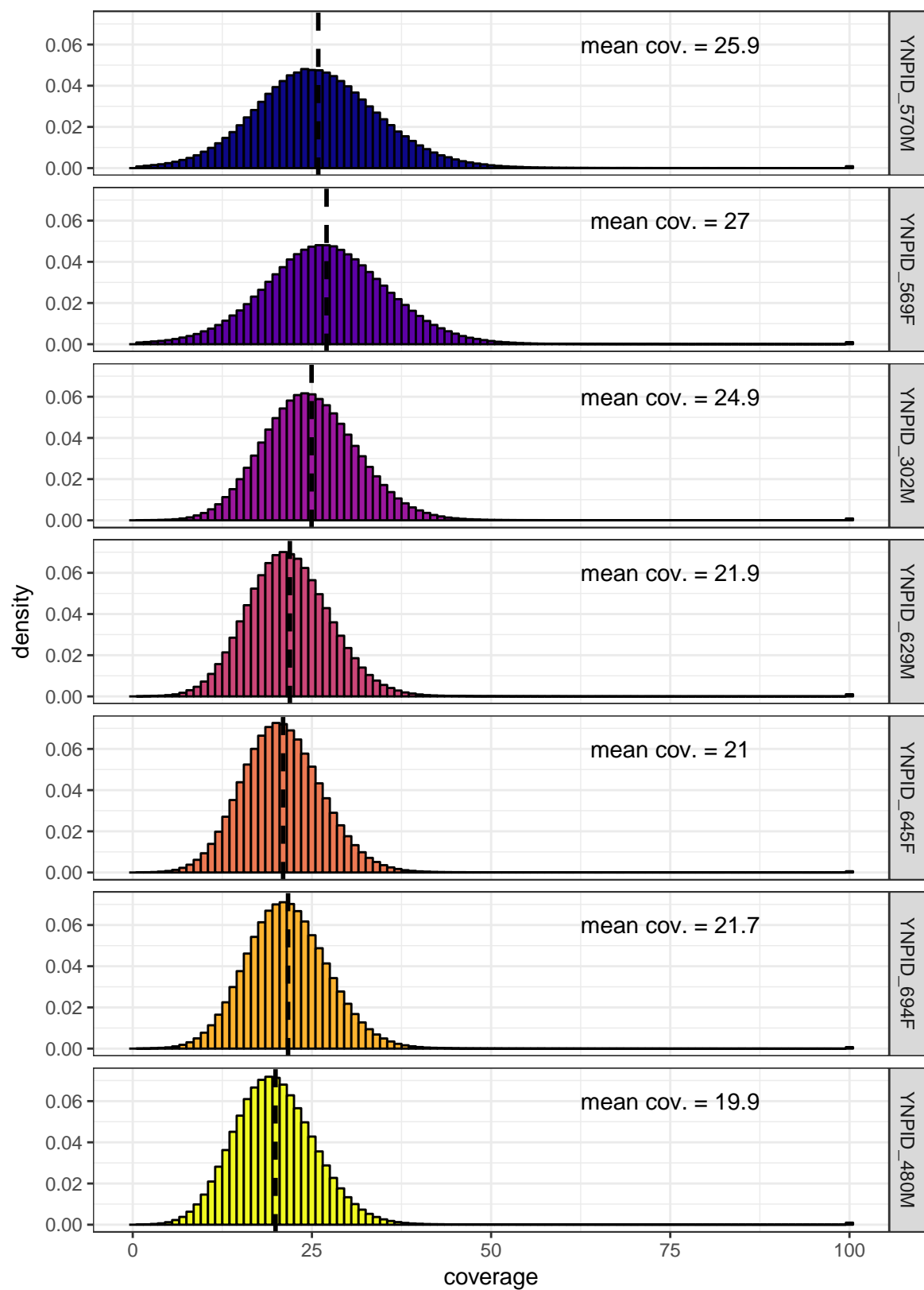

Supplement: msz159_Supplementary_Data [file msz159_supplementary_data.zip › figure_s2.pdf]

$(DN_p < 0.3) \times (\text{prop. at depth})$

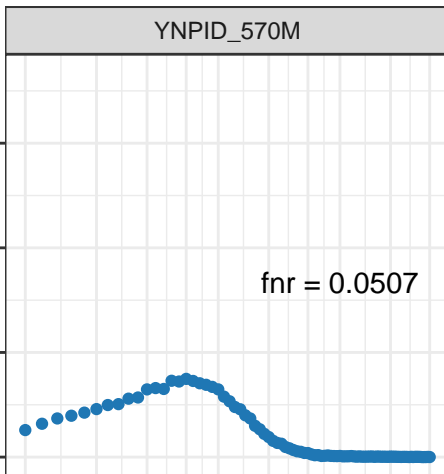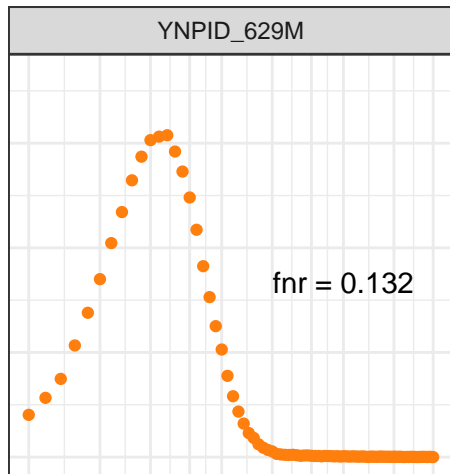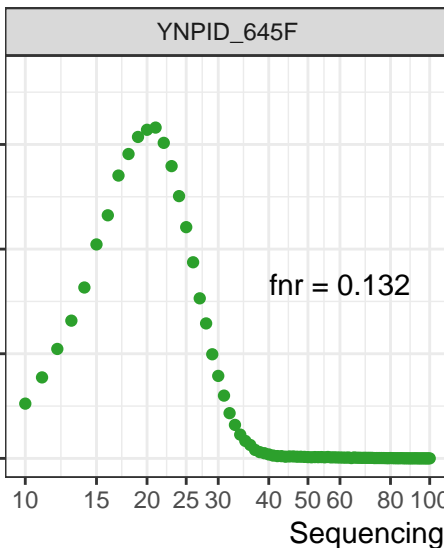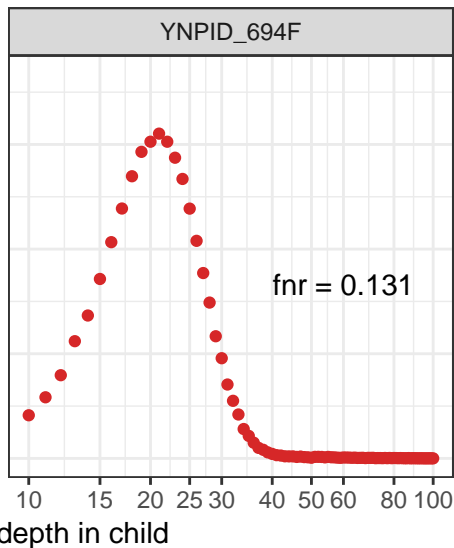

Sequencing depth in child

Supplement: msz159_Supplementary_Data [file msz159_supplementary_data.zip › figure_s3.pdf]

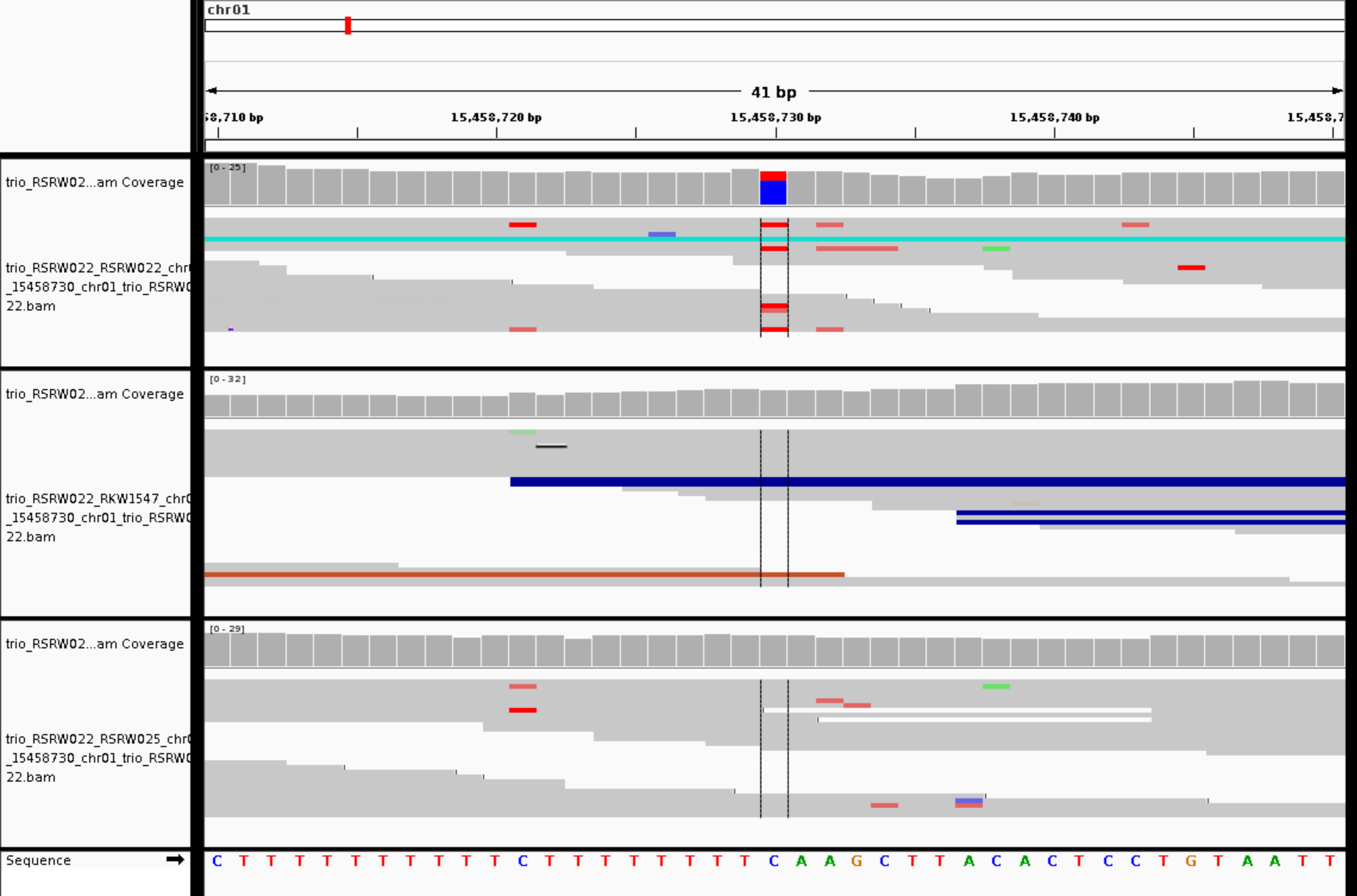

Supplement: msz159_Supplementary_Data [file msz159_supplementary_data.zip › figure_s5.pdf]

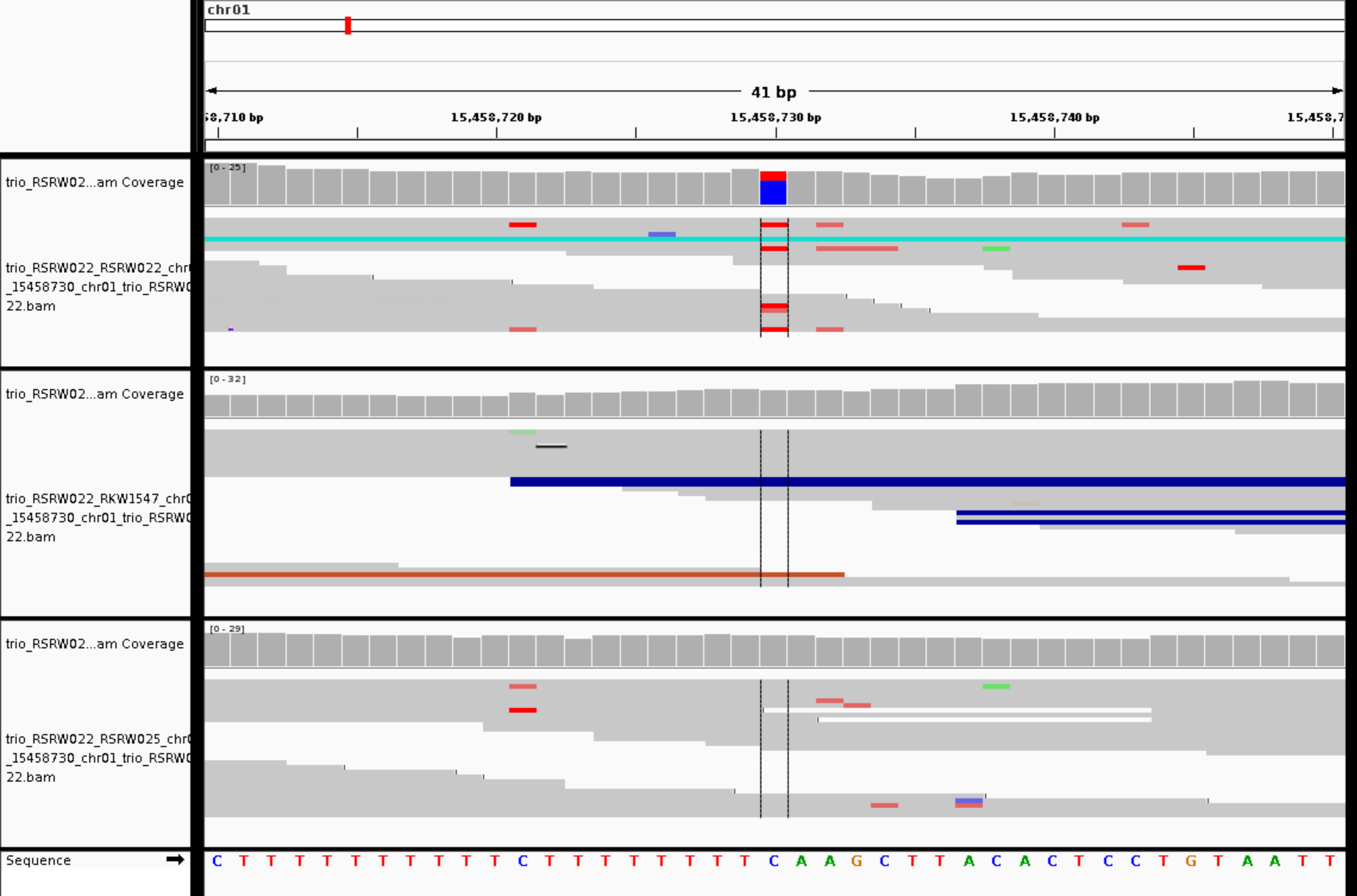

Supplement: msz159_Supplementary_Data [file msz159_supplementary_data.zip › figure_s6.pdf]

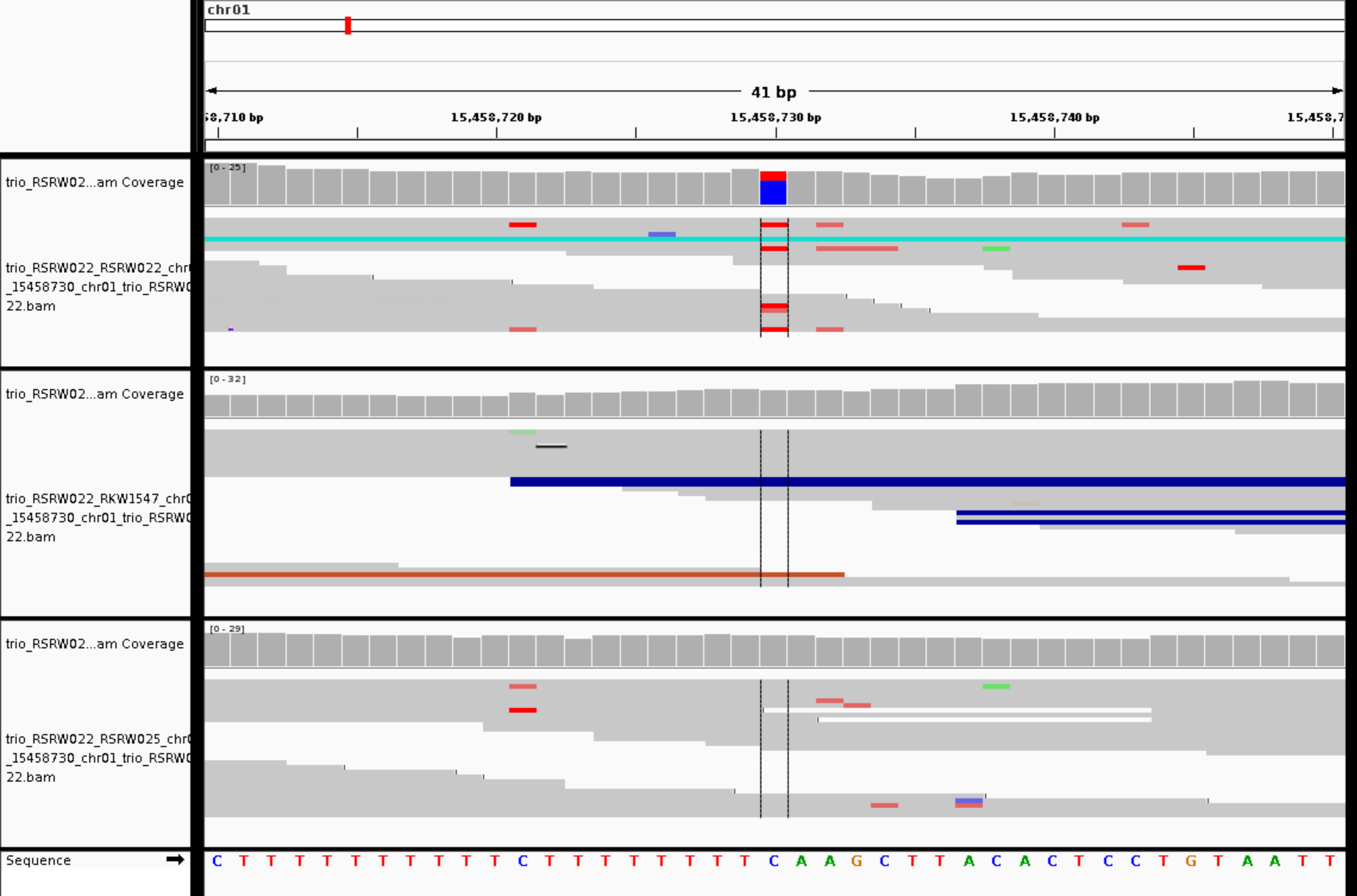

Supplement: msz159_Supplementary_Data [file msz159_supplementary_data.zip › figure_s7.pdf]

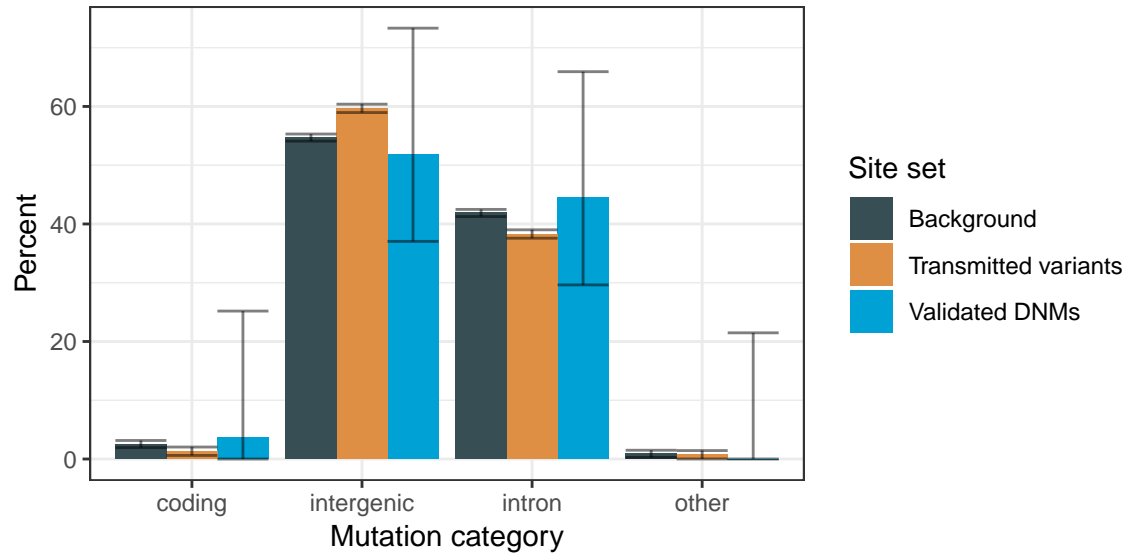

Supplement: msz159_Supplementary_Data [file msz159_supplementary_data.zip › figure_s8.pdf]

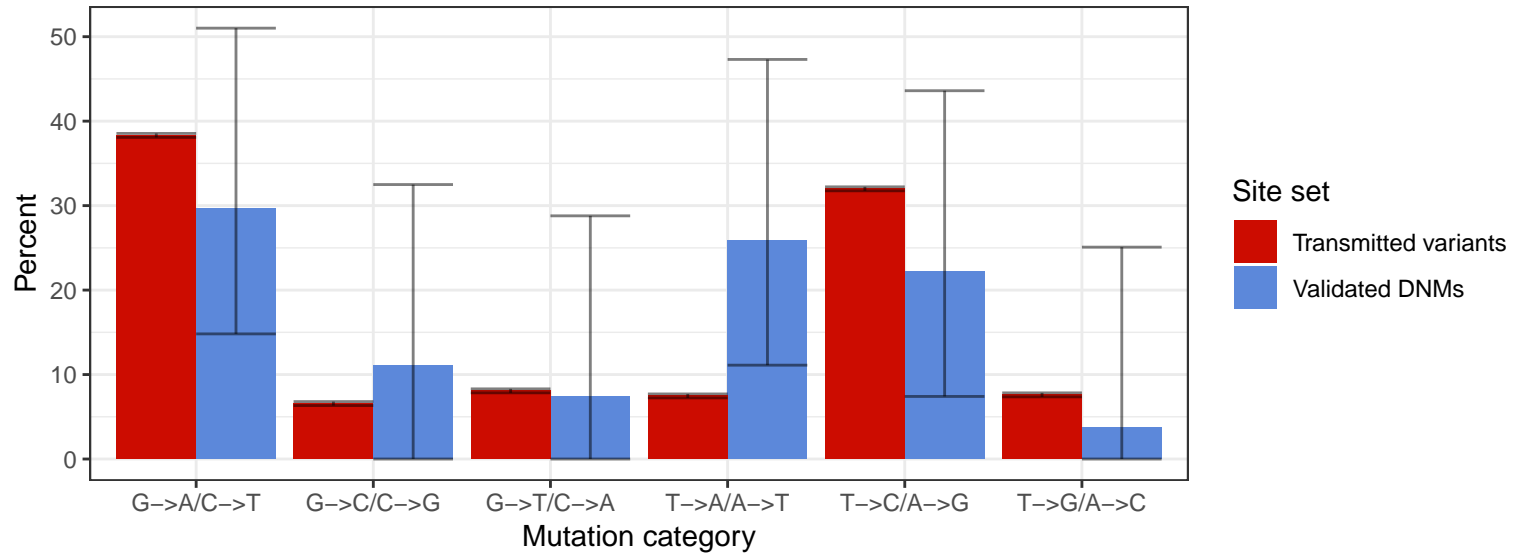

Supplement: msz159_Supplementary_Data [file msz159_supplementary_data.zip › figure_s9.pdf]
